# Editorial: New insights into social isolation and loneliness

**DOI:** 10.3389/fpsyt.2024.1401798

**Published:** 2024-03-26

**Authors:** Yuka Kotozaki, Lené Levy-Storms

**Affiliations:** ^1^ Iwate Medical University, Yahaba, Japan; ^2^ University of California, Los Angeles, Los Angeles, CA, United States

**Keywords:** social support, loneliness, pandemic, social isolation, stigma, mental health

This Research Topic provides new insights into social isolation and loneliness. Social isolation represents a significant public health problem, with well-documented detrimental consequences for people’s health, including reduced mental well-being, an increased risk of diseases such as hypertension, cardiovascular disease, cancer, mortality, and cognitive decline. Ample evidence supports that social isolation is a major contributor to mortality. With the recent impact of COVID-19 and other changes in social conditions, public health leaders have underscored how social isolation and loneliness have become increasingly concerning. Social isolation can affect individuals regardless of gender or age, and further investigation will provide understanding about the occurrence process and related factors. This Research Topic aims to enhance our understanding of social isolation and loneliness.

Ten out of the 17 manuscripts submitted to the journal by international researchers were deemed suitable for publication after undergoing a thorough peer review process. The following is a summary of the main results for each manuscript.

In the first article in this special Research Topic, Babalola et al. explored the determinants of social support among Nigerians living with HIV. In a cross-sectional study in Lagos State, Nigeria in 2021, 400 persons living with HIV from six health facilities responded to surveys about perceived social support and HIV stigma. The sample reported substantive social support in general, but stigma was negatively associated with social support. However, being female, higher income, and disclosing seropositive status positively associated with social support. HIV-related stigma negatively affects social support, but support from family and friends reduced this effect. The implications call for more social support in this vulnerable population.

In the next article, Fan et al. examined the mediating effect of stigma between self-perceived burden and loneliness in stroke patients. This study found a positive correlation between loneliness in stroke patients and their self-perceived burden and stigma. Mediation analysis indicated that stigma played a complete mediating role in the relationship between self-perceived burden and loneliness. Overall, the results emphasize the significance of stigma as a crucial modifiable psychological factor influencing the loneliness of stroke patients.

In the third article of this Research Topic, Holm-Hadulla et al. described the depression and social isolation during the COVID-19 pandemic in a student population. The survey, involving 27,162 participants, utilized the Patient Health Questionnaire (PHQ) and the Well-Being Index WHO-5. Findings revealed that after 1.5 years of restrictions, 40.16% reported “major” depressive syndromes, and 72.52% experienced severely reduced well-being. Nine months post-restrictions, “major” depressive syndromes decreased to 28.50%, and well-being improved (53.96% with a Well-Being Index below 50). The study indicated a link between depressive syndromes and reduced well-being with social isolation and loneliness. Concerns about “loneliness and social isolation” decreased from 24.2% during restrictions to 7.7% after 9 months of eased restrictions. Qualitative analysis suggested a shift towards actively addressing loneliness, potentially contributing to the later reduction in depressive syndromes.

Yuan et al. examined the relationship between fall and loneliness among older people in China. A survey involving 4,289 older individuals identified significant differences in loneliness based on age, marital status, education, residence, solitariness, and falls. The study revealed that falls, especially occurring once, contributed to increased loneliness in older individuals. Notably, agreeableness, conscientiousness, and neuroticism played significant mediating roles between falls and loneliness. The findings suggest that considering the big five personality traits is crucial for understanding and addressing loneliness in older individuals.

Andrade et al. examined impact of social isolation caused by the COVID-19 pandemic on the mood profile of active and sedentary older adults. This observational study during the COVID-19 pandemic in southern Brazil focused on older adults over 60. Using an online questionnaire in May 2020 and June 2021, 150 participants were surveyed about sociodemographics, physical activity (PA), confinement, and mood states. Of these, 53.3% reported engaging in PA. Active older adults showed fewer mood changes, experiencing lower levels of confusion, depression, and fatigue compared to inactive peers. Prolonged confinement (over 50 days) correlated with a higher risk of depression. Mood states were influenced by the fear of contracting COVID-19, with greater fear associated with more mental confusion, depression, fatigue, tension, and lower vigor. Additionally, the study highlighted a positive correlation between the hours dedicated to PA and improved mood states, indicating potential benefits for older adults.

Wenig et al. examined the associations of loneliness with mental health and with social and physical activity among university students in Germany. The COVID-19 German Student Well-Being Study (C19 GSWS) conducted between October 27th and November 14th, 2021, gathered data from 7,203 respondents across five German universities. Loneliness, reported by 20.6% of students, was analyzed in relation to depressive symptoms, anxiety, physical and social activity, and sociodemographic characteristics. Students with depressive or anxiety symptoms had significantly higher odds of reporting loneliness. Less physical activity was associated with increased loneliness, while no association was found with social activity. Loneliness was linked to being single, living alone, and having temporary residency status in Germany.

Yong conducted a secondary data analysis from a 2012 internet addiction survey to understand nuanced factors associated with “hikikomori” or extreme social withdrawal. In particular, the study focused on outgoing behaviors from hikikomori and their association with loneliness. Factor analyses on a sample of 623 Japanese internet users found mental health factors like stress, distress, dissatisfaction with personal life were strongly associated with loneliness. These results contrast with usual classifications of hikikomori and suggest a need for a reevaluation of hikikomori among individuals working or pursuing education.

Sipowicz et al. aimed to evaluate the occurrence and severity of reactive depressive episodes, loneliness, and the sense of meaning in life in individuals who had a pulmonary SARS-CoV-2 infection about a year earlier. The participants included 63 hospitalized, 67 non-hospitalized patients, and 60 healthy controls. Hospitalized patients exhibited the highest frequency and severity of depression, followed by non-hospitalized individuals, and both groups showed significant differences compared to healthy controls. Feelings of loneliness were most pronounced in the hospitalized group, and loneliness severity was higher in outpatients compared to the control group. The sense of meaning in life was lowest among hospitalized patients, moderately reduced in outpatients, and typical of the general Polish population in the control group.

Finally, Jin and Hwang examined the neuro-cognitive deficits associated with loneliness in young adults. Two groups, high-lonely and low-lonely, were identified based on the UCLA Loneliness Scale v.3. The high-lonely group exhibited significantly poorer executive function and attention, even after accounting for depression and anxiety. The study suggests that loneliness may initially affect executive function and attention in early adulthood, later extending to other cognitive domains, similar to findings in the elderly. The results also indicate that depression and anxiety do not mediate the relationship between loneliness and neuro-cognitive functioning.

In conclusion, the editors wish to thank all the authors, the reviewers, and the editorial board members for contributing to this Research Topic. Loneliness represents a complex social problem transcending demographics, health, geography, and cultures. We hope this Research Topic might inspire future and novel research approaches in the field of social isolation and loneliness.

## Author contributions

YK: Writing – original draft, Writing – review & editing. LL-S: Writing – original draft, Writing – review & editing.

